# Percutaneous stenting for multifaceted fibrosing mediastinitis with multivessel involvement

**DOI:** 10.1093/ehjcr/ytaf077

**Published:** 2025-02-14

**Authors:** Meiyan Zhao, Hongling Su, Yunshan Cao

**Affiliations:** The First Clinical Medical College of Gansu University of Chinese Medicine (Gansu Provincial Hospital), No. 35, Dingxi East Road, Chengguan District, Lanzhou, Gansu 730000, China; Department of Cardiology, Pulmonary Vascular Disease Center, Gansu Provincial Hospital, No. 204, Donggang West Road, Chengguan District, Lanzhou, Gansu 730000, China; Heart, Lung and Vessels Center, Sichuan Provincial People’s Hospital, University of Electronic Science and Technology of China, No. 32, West Second Section, 1st Ring Road, Qingyang District, Chengdu, Sichuan 610072, China

A 72-year-old woman with exertional dyspnoea and facial oedema for 8 months was referred to our centre. Echocardiography suggested pulmonary hypertension (PH). To further clarify the cause of PH, contrast-enhanced computed tomography was performed and revealed soft tissue proliferation in the mediastinum (*Panel A*), encasing and compressing the superior vena cava (SVC) (*Panel B*), pulmonary artery (PA) (*Panel C*), pulmonary vein (PV) (*Panel D*), and bronchi (*Panel E*). Therefore, the patient was diagnosed with fibrosing mediastinitis (FM). Angiography revealed severe stenosis in the middle segment of the SVC (*Panel F*) and in the left lower lobe of the PA (*Panel G*). After multiple balloon dilatations, a bare-metal stent (10 × 40 mm, Boston Scientiﬁc Carotid WALLSTENT™) was successfully implanted into the SVC (*Panel H*; Supplementary material online, *Video S1*). One month later, a bare-metal stent (7 × 19 mm, Boston Scientiﬁc Express™ Vascular SD) was implanted in the basal trunk of the left lower lobe PA (*Panel I*; Supplementary material online, *Video S2*). The patient experienced significant improvement in symptoms immediately after stenting and was able to resume daily activities.

**Figure:**
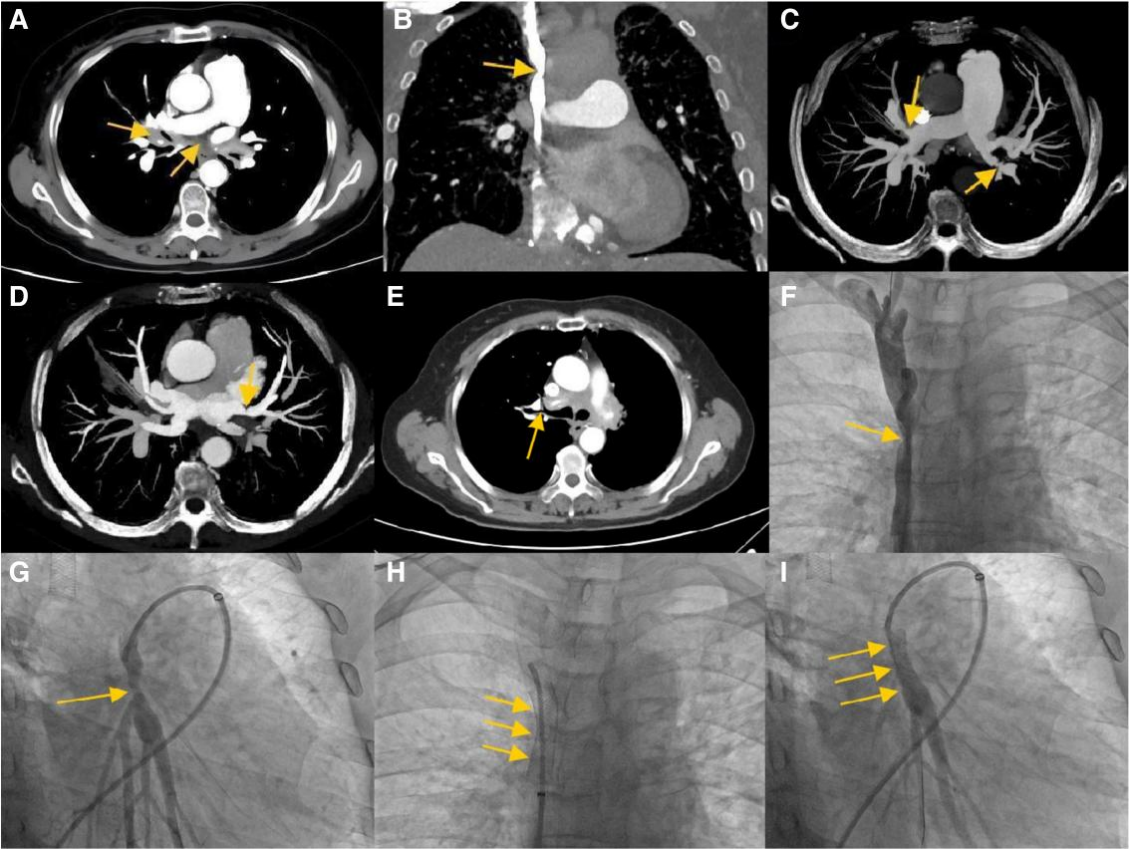


Fibrosing mediastinitis is a rare disease characterized by the proliferation of fibrous tissue within the mediastinum; however, the pathogenesis of FM is still unclear, and the most common triggers are *Histoplasma capsulatum* and *Mycobacterium tuberculosis* infections. Symptoms in patients with FM are diverse and depend on the structures involved in the mediastinum, including the PA, PV, SVC, bronchi, oesophagus, pericardium, and thoracic ducts, with corresponding symptoms of dyspnoea and haemoptysis, pleural effusion, SVC syndrome, pulmonary atelectasis and pneumonia, dysphagia, constrictive pericarditis, and chylothorax disease. However, FM rarely shows simultaneous involvement of multiple structures, such as the PA, PV, bronchus, and SVC, as reported in this case. Depending on the site of involvement, different treatments should be chosen. Due to the poor effects of drugs and surgical treatment, percutaneous vascular stent implantation is an effective method for treating FM. Given the diversity of symptoms in FM patients, FM should be considered when patients have dyspnoea, pleural effusion, atelectasis, SVC syndrome, and other clinical manifestations including FM dyad, FM triad, and Yunshan sign, and further imaging examination should be performed to avoid misdiagnosis and missed diagnosis.

## Supplementary Material

ytaf077_Supplementary_Data

## Data Availability

The data underlying this article are available in the article and in its online Supplementary material.

